# Renalase Attenuates Mouse Fatty Liver Ischemia/Reperfusion Injury through Mitigating Oxidative Stress and Mitochondrial Damage via Activating SIRT1

**DOI:** 10.1155/2019/7534285

**Published:** 2019-12-14

**Authors:** Tao Zhang, Jian Gu, Jianrong Guo, Ke Chen, Huili Li, Jiliang Wang

**Affiliations:** Department of Gastrointestinal Surgery, Union Hospital, Tongji Medical College, Huazhong University of Science and Technology, Wuhan 430022, China

## Abstract

Liver ischemia/reperfusion (IR) injury is a severe complication of liver surgery. Moreover, nonalcoholic fatty liver disease (NAFLD) patients are particularly vulnerable to IR injury, with higher rates of postoperative morbidity and mortality after liver surgeries. Our previous study found that renalase (RNLS) was highly sensitive and responsive to oxidative stress, which may be a promising biomarker for the evaluation of the severity of liver IR injury. However, the role of RNLS in liver IR injury remains unclear. In the present study, we intensively explored the role and mechanism of RNLS in fatty liver IR injury in vivo and in vitro. C57BL/6 mice were divided into 2 groups feeding with high-fat diet (HFD) and control diet (CD), respectively. After 20 weeks' feeding, they were suffered from portal triad blockage and reflow to induce liver IR injury. Additionally, oleic acid (OA) and *tert*-butyl hydroperoxide (t-BHP) were used in vitro to induce steatotic hepatocytes and to simulate ROS burst and mimic cellular oxidative stress following portal triad blockage and reflow, respectively. Our data showed that RNLS was downregulated in fatty livers, and RNLS administration effectively attenuated IR injury by reducing ROS production and improving mitochondrial function through activating SIRT1. Additionally, the downregulation of RNLS in the fatty liver was mediated by a decrease of signal transduction and activator of transcription 3 (STAT3) expression under HFD conditions. These findings make RNLS a promising therapeutic strategy for the attenuation of liver IR injury.

## 1. Introduction

Liver ischemia/reperfusion (IR) injury is the phenomenon in which hepatocellular damage in the ischemic liver is aggravated after the restoration of blood flow [1]. Liver IR injury is a severe complication commonly associated with liver surgery, including liver resection and transplantation, which leads to liver failure or primary nonfunction [2]. Nonalcoholic fatty liver disease (NAFLD) is defined as the 5-10% steatosis of hepatocytes due to nonalcoholic causes [3], and this disease is one of the most rapidly increasing and important causes of liver disease, affecting approximately 20% of the general population and up to 70% of type 2 diabetes patients [4]. Liver steatosis increases the risk of postoperative morbidity and mortality after liver surgery because steatotic livers are particularly vulnerable to IR injury [5, 6]. However, the molecular mechanisms underlying the susceptibility of IR injury in patients with NAFLD remain unclear. Since liver resection and transplantation are the most effective therapeutic methods for end-stage liver diseases, such as liver cancer and liver cirrhosis, the impact of steatosis is increasingly considered in liver surgery due to the increasing obese population. Thus, it is of vital importance to investigate the underlying mechanisms and possible preoperative and perioperative interventions for minimizing IR-induced hepatocellular damage, especially in NAFLD patients.

The excessive production of ROS and subsequent disorder of redox balance are the most invoked mechanisms in liver IR injury [7]. During liver IR, hepatic sinusoidal endothelial cells, vascular endothelial cells, Kupffer cells, or neutrophils are activated and produce a large amount of ROS, including superoxide, hydrogen peroxide (H_2_O_2_), hydroxyl radicals, and nitric oxide [7]. In addition, xanthine oxidase, nicotinamide adenine dinucleotide phosphate (NADPH) oxidase, mitochondria, inducible nitric oxide synthase (iNOS), cytochrome P450, lipoxygenase, and monoamine oxidase are the major intracellular sources of ROS during IR [8]. Mitochondria have been implicated as the main source as well as the first target of the generated ROS in IR. The excessive production of ROS leads to mitochondrial membrane peroxidation and the collapse of mitochondrial membrane potential (*ΔΨ*), leading to the failure of ATP synthesis, the release of cytochrome C, and consequent irreversible cellular injury or death [9]. Moreover, damaged mitochondria will generate more ROS. Thus, attenuating the generation of ROS and subsequent mitochondrial dysfunction to block the vicious “ROS-mitochondria-ROS” cycle may be a vital therapeutic mechanism to attenuate liver IR injury.

Renalase (RNLS), a ubiquitous flavin adenine dinucleotide-dependent amine oxidase, is synthesized in various organs, including the kidneys, heart, liver, and adipose tissues [10]. RNLS exerts enzymatic activity that catalyzes oxidation-reduction processes in some primary metabolic pathways, including catecholamine metabolism. Additionally, RNLS also functions as a cytokine that activates the PI3K/AKT and ERK signaling pathways and increases cell survival in a manner completely independent of its enzymatic properties [11]. RNLS has been implicated in IR injury and exerts a protective role against IR injury in the heart [12, 13] and kidney [14]. In addition, our previous study found that RNLS was highly sensitive and responsive to oxidative stress, which may be a promising biomarker for the evaluation of the severity of liver IR injury [15]. However, the role of RNLS in liver IR injury remains unclear. Additionally, RNLS specifically oxidizes *α*-reduced nicotinamide adenine dinucleotide (*α*-NADH) and converts this compound into *β*-nicotinamide adenine dinucleotide (*β*-NAD^+^) [16]. NAD^+^ is critically important for energy metabolism and indispensable for the activity of the NAD^+^-dependent class III histone deacetylase sirtuin 1 (SIRT1) [17]. SIRT1 deacetylates various target proteins and is consequently involved in a variety of pathophysiological processes, such as antiaging, oxidative resistance, antiapoptosis, and anti-inflammation [18], which play a protective role in IR injury [19, 20].

In this study, we hypothesized that RNLS protects against liver IR injury by increasing the NAD^+^ content to promote SIRT1 activity. Our data, to our knowledge, is the first to demonstrate that RNLS downregulated liver steatosis in vivo and in vitro, and exogenous RNLS supplementation protected against liver IR injury through mitigating oxidative stress and improving mitochondrial function. Our results provide evidence that RNLS plays a role in protecting against liver IR injury, and the downregulation of RNLS leads to the susceptibility of fatty livers to IR injury.

## 2. Materials and Methods

### 2.1. Ethics Statement

All experimental procedures were performed in accordance with the International Guidelines for the Care and Use of Laboratory Animals and approved by the Animal Ethical Committee of Tongji Medical College, Huazhong University of Science and Technology.

### 2.2. Synthesis of Recombinant Human RNLS

Human recombinant RNLS was provided by Sangon Biotech (Shanghai, China), which synthesized through a prokaryotic expression system. Briefly, the gene sequence of human RNLS was subjected to codon optimization to facilitate expression in E. coli. Untagged, recombinant amino acids 1 to 342 of RNLS were generated by cloning the coding region into the NdeI/XhoI sites of the pET28a vector. Then, Rosetta (DE3) was transformed with recombinant vectors and grown overnight in Luria-Bertani (LB) medium containing kanacillin and chloromycetin. After transformation, the positive clone plasmids were selected and grown in LB medium containing kanacillin and chloromycetin. When the optical density (OD) value comes to 0.6, 0.5 mM isopropyl *β*-D-1-thiogalactopyranoside (IPTG) was added to the medium and then cultured overnight. After culturing, the bacteria were collected and dissolved in 10 mL of solubilization buffer (8 M urea, 50 mM Tris, 300 mM NaCl, 0.1% Triton X-100, and pH 8.0) by ultrasonication for 20 min. Then, the solution was centrifuged at 12000 rpm for 20 min, and the supernatant was collected for further purification through nickel-sepharose affinity chromatography to 90% homogeneity finally.

NADH oxidase activity was used for testing the activity of recombinant RNLS according to the manufacturer's protocols. Briefly, after treatment with or without recombinant RNLS for 3 h, cells were lysed with 200 *μ*L cold NAD^+^/NADH extract solution. After centrifugation at 12000 g for 10 min, the supernate was collected for testing. For NAD_total_ measurement, 20 *μ*L of samples was added to a 96-well plate, and then, 90 *μ*L alcohol dehydrogenase (ADH) was added to the plate. For NADH measurement, samples were first incubated in 60°C for 30 min to resolve NAD^+^. After that, 20 *μ*L of samples was added to a 96-well plate, and then, 90 *μ*L alcohol dehydrogenase (ADH) was added to the plate. After incubating in 37°C for 10 min, 10 *μ*L color substrate solution was added to the mixture and then incubated in 37°C for 30 min. Finally, the optical density (OD) was measured by a microplate reader at 450 nm (NAD^+^ = NAD_total_–NADH).

The results of RNLS synthesis and activity of recombinant RNLS were shown in Supplementary Figures [Supplementary-material supplementary-material-1] and [Supplementary-material supplementary-material-1].

### 2.3. Reagents

OA, *tert*-butyl hydroperoxide (t-BHP), NAD^+^, and Oil Red O were purchased from Sigma-Aldrich (St. Louis, MO, USA). In addition, 2′,7′-dichlorofluorescin diacetate (DCFH-DA) and TRIzol reagent were purchased from Invitrogen, Thermo Fisher Scientific (Carlsbad, CA, USA). A NAD^+^/NADH assay kit with WST-8, a mitochondrial membrane potential assay kit with JC-1, a cell mitochondria isolation kit, a lipid peroxidation malondialdehyde (MDA) assay kit, a BCA protein assay kit, a reduced glutathione (GSH) and oxidized glutathione (GSSG) assay kit, a terminal deoxynucleotidyl transferase dUTP nick end labeling (TUNEL) staining kit, a total superoxide dismutase (SOD) assay kit with WST-8, an ATP Assay Kit, and EX527 were purchased from Beyotime (Hangzhou, China). Radioimmunoprecipitation assay (RIPA) buffer and PMSF were purchased from BOSTER Biological Technology (Wuhan, China). HiScript® II Q RT SuperMix for qPCR (+gDNA wiper) and ChamQ™ SYBR® qPCR Master Mix (high 5-carboxy-X-rhodamine, succinimidyl ester (ROX) premixed) were purchased from Vazyme Biotech Co. Ltd. (Nanjing, China). Alanine aminotransferase (ALT), aspartate aminotransferase (AST), a lactate dehydrogenase (LDH) kit, a total cholesterol (TC) assay kit, and a triglyceride (TG) assay kit were purchased from Nanjing Jiancheng (Nanjing, China). MitoSOX Red Mitochondrial Superoxide Indicator was purchased from YEASEN (Shanghai, China). For western blotting, antibodies against GAPDH, SIRT1, Bax, and Bcl2 were purchased from BOSTER Biological Technology; anti-RNLS was purchased from Abcam (Cambridge, UK); and antibodies against Drp1 and cyto C were purchased from Cell Signaling Technology Inc. (Beverly, MA, USA). A mouse RNLS ELISA kit was purchased from Bio-Swamp (Wuhan, China).

### 2.4. Cell Culture and Treatment

Human hepatocellular carcinoma cell line HepG2 was obtained from the Cell Bank of the Chinese Academy of Sciences (Shanghai, China) and cultured in DMEM medium (Gibco; Thermo Fisher Scientific, Inc., Waltham, MA, USA) with 10% fetal bovine serum (FBS) (ScienCell Research Laboratories, Inc., San Diego, CA, USA) and 1% penicillin/streptomycin (Gibco; Thermo Fisher Scientific, Inc.). In addition, the cells were cultured at 37°C in an incubator with 5% CO_2_.

To induce steatosis, HepG2 cells were incubated in OA medium (OA was diluted with dimethyl sulfoxide (DMSO) and added to DMEM supplemented with 10% FBS) for 24 h when the HepG2 cells were approximately 80% confluent. The cells incubated in the same concentration of DMSO were used as a negative control group (Ctrl group). To induce oxidative stress, HepG2 cells were exposed to t-BHP in serum-free DMEM for 3 h.

### 2.5. Transfection

A lentivirus vector containing the RNLS-siRNA sequence (si-RNLS1# and 2#) or a negative control (si-NC) was purchased from GeneChem (Shanghai, China). To establish a stable cell line with RNLS downregulation, lentiviral vectors with RNLS-siRNAs were transfected into HepG2 cells according to the manufacturer's protocols. Briefly, HepG2 cells were incubated in enhanced infection solution with polybrene and lentivirus. After 8 h, the mixture was changed with DMEM medium with 10% FBS for 3 days; the transfection efficiency was identified via observing fluorescence intensity.

Si-STAT3 and matched si-NC were purposed from RiboBio (Guangzhou, China) and transfected using Lipofectamine 3000 (Invitrogen, Thermo Fisher Scientific, Inc.) according to the manufacturer's protocols as previously described [21]. Briefly, HepG2 cells were in Opti-MEM (Gibco, Thermo Fisher Scientific, Inc.) with premixed Lipofectamine 3000 and si-STAT3 for 3 h. Then, the mixture was changed with DMEM medium with 10% FBS for 48 h. The sequences of siRNAs are shown in Supplementary [Supplementary-material supplementary-material-1].

### 2.6. Cell Viability

Cell viability was determined by the MTT assay. Briefly, 1 × 10^4^ cells/per well were seeded onto 96-well plates and incubated for 24 h at 37°C. To detect the cytotoxic effects of OA, DMSO, t-BHP, RNLS, NAD^+^, and EX527, the cells were treated with various concentrations of OA (24 h), DMSO (24 h), t-BHP (3 h), RNLS (4 h), NAD^+^ (4 h), and EX527 (4 h). To evaluate the dose-dependent protective effects of RNLS or NAD^+^ against t-BHP-induced cytotoxicity, HepG2 cells were pretreated with biologically safe concentrations of RNLS or NAD^+^, and then, cytotoxic concentrations of t-BHP were added to the medium containing RNLS or NAD^+^ for 3 h. After treatment, 20 *μ*L of MTT (5 mg/mL) in PBS solution was added to medium and incubated for 4 h. Then, the medium was carefully removed, and 150 *μ*L of DMSO was added in each well to solubilize the crystals. Finally, the optical density (OD) was measured by a microplate reader at 490 nm. All viability assays were performed in duplicate; the percentage growth inhibition was calculated using the following formula: Cell viability (%) = (*A*_490 treatment_‐*A*_490 blank_)/(*A*_490 control_‐*A*_490 blank_) × 100 (control: serum-free DMEM with cells; blank: serum-free DMEM without cells).

### 2.7. Cell Oil Red O Staining

Oil Red O stock solution was made by combining 0.2 g Oil Red O with 40 mL isopropanol. The Oil Red O working solution was made fresh on the day of staining by diluting the stock solution with ddH_2_O at a ratio of 3 : 2, and the working solution was then filtered before use. HepG2 cells were treated with OA as described above. After treatment, the cells were washed with sterile PBS, fixed with 4% paraformaldehyde for 30 min, and stained with Oil Red O working solution for 30 min at room temperature. The Oil Red O solution was then removed, and the wells were washed 3 times with PBS to remove the excess stain. Finally, the nucleus was stained with hematoxylin solution for 30 sec and then rinsed with tap water. The stained lipid droplets within the cells were visualized and photographed using an inverted microscope (Olympus, Tokyo, Japan).

### 2.8. Measurement of the TG and TC Levels

The TG and TC levels were quantified by using a commercial kit according to the manufacturer's protocol. For intracellular TG measurement, after OA treatment, the cells were rinsed with PBS and then homogenized and sonicated in lysis buffer on ice. The TG levels in the cell lysate were measured. The protein concentration of each sample was determined by a BCA protein assay kit. In addition, TG levels were normalized according to the protein concentrations. For the TG and TC measurements of liver tissues, liver tissues were harvested and rinsed with PBS and then homogenized and sonicated in lysis buffer on ice after surgery. The TG and TC levels in the tissue lysate were measured and normalized by the protein concentrations.

### 2.9. Measurement of the Cell Total and Mitochondrial ROS Levels

Cell total ROS produced in the cultured cells were stained with DCFH-DA. After t-BHP treatment, DCFH-DA at a final concentration of 10 *μ*M diluted in DMEM was added to each well and incubated at 37°C for 30 min in the dark. The fluorescence was then measured with flow cytometry and fluorescence microscopy. Additionally, MitoSOX Red Mitochondrial Superoxide Indicator was used for mitochondrial ROS measurement. After t-BHP treatment, the indicator at a final concentration of 5 *μ*M in diluted in DMEM was added to each well and incubated at 37°C for 10 min in the dark. The fluorescence was then measured with fluorescence microscopy. The MitoSOX fluorescence intensity was analyzed by ImageJ software.

### 2.10. Measurement of the Mitochondrial Membrane Potential (*ΔΨ*m)

The alteration of *ΔΨ*m in HepG2 cells was analyzed using a JC-1 staining assay kit according to the manufacturer's instructions. Briefly, after t-BHP treatment, the cells were then rinsed with PBS and stained with JC-1 (20 *μ*g/mL) at 37°C in the dark for 30 min. After two rinses with staining buffer, the fluorescence was then detected by flow cytometry and fluorescence microscopy.

### 2.11. Animals

Male C57BL/6 mice (6 weeks old) were purchased from Beijing Huafukang Bioscience Co. Inc. (Beijing, China) and housed in the Animal Care Facility of Tongji Medical College at 25°C, 45–75% relative humidity, and a 12 h light-dark cycle. After a 1-week adaptive phase, all mice were randomly divided into two groups: one group was fed a regular control diet (CD) (10% fat), and the other group was fed a HFD (60% fat) for 20 weeks to develop fatty livers.

### 2.12. In Vivo Hepatic IR Model

The mice were divided into 6 groups: CD-sham, CD-IR, CD-IR+RNLS, HFD-sham, HFD-IR, and HFD-IR+RNLS, with 5 mice in each group. The surgical procedures were performed as previously described to induce hepatic IR injury in 70% of the liver [22]. Fasted mice were anesthetized with pentobarbital sodium (50 mg/kg) by an intraperitoneal injection and subjected to a midline incision to expose the liver. An atraumatic clamp was placed across a branch of the portal triad to block the blood supply to the median and left lateral liver lobes to induce ischemia for 90 min. Following unclamping of the liver, hepatic reperfusion was performed for 2 h, and this procedure represented the IR group. In the RNLS group, RNLS recombinant protein (0.5 mg/kg) was injected through the tail vein at 15 min before and after IR operation as previously described [12]. In the sham group, the same surgical procedure was performed but without clamping. During the surgical operation, 0.1 mL of 25 units/mL heparin was injected into all mice to prevent thrombosis prior to the IR procedure. Following a 2 h reperfusion period, blood was collected from the inferior vena cava. The harvested liver tissues were frozen in liquid nitrogen, stored at -80°C, and subsequently fixed in formalin for histology and immunohistochemistry (IHC).

### 2.13. Measurement of Liver Injury Markers

After collecting serum samples from the mice, serum levels of ALT, AST, and LDH were detected using commercially available kits according to the manufacturer's introductions. Briefly, for ALT and AST measurements, 5 *μ*L serum sample was added to 20 *μ*L kubstrate for ALT or AST. After incubating for 30 min in 37°C, 20 *μ*L color substrate solution was added to the mixture and incubated in 37°C for 20 min. And the 200 *μ*L stop buffer was then added and stopped the reaction in room temperature for 15 min. Finally, the OD was measured by a microplate reader at 510 nm. For LDH measurement, serum samples were added to the mixture combined with 25 *μ*L matrix buffer and 5 *μ*L coenzyme I. After incubating for 15 min in 37°C, 25 *μ*L 2,4-dinitrophenylhydrazine was added and incubated for another 15 min in 37°C. Finally, 250 *μ*L 0.4 mol/L NaOH was added to the mixture incubating for 5 min, and the OD was measured by a microplate reader at 450 nm.

### 2.14. Measurement of the MDA, GSH, and SOD Levels

The MDA, GSH, and SOD were quantified by using commercial kits according to the manufacturer's protocol. For MDA and SOD measurement, the liver tissues were rinsed with PBS and then homogenized and sonicated in lysis buffer on ice. After sonication, the lysed tissues were centrifuged at 10000 g for 10 min to remove debris. For GSH measurement, liver tissues were homogenized through three freeze-thaw cycles, and then, the tissue suspension was centrifuged at 12000 rpm for 5 min at 4°C. The MDA, GSH, and SOD levels in the supernatant were measured. The protein concentration of each sample was determined by a BCA protein assay kit. In addition, MDA, GSH, and SOD levels were normalized according to the protein concentrations.

### 2.15. Measurement of Mitochondrial DNA (mtDNA)

Mitochondrial DNA (mtDNA) was extracted from liver tissue using a DNeasy Blood & Tissue kit (Qiagen, Valencia, CA, USA). Real-time PCR was performed via the Applied Biosystems StepOne Real-Time PCR system using ChamQ™ SYBR® qPCR Master Mix (High ROX Premixed) containing 5 ng cDNA and 10 pM of each primer. The cycling conditions consisted of one cycle at 95°C for 30 sec, 40 cycles at 95°C for 10 sec, and 60°C for 30 sec. A melting curve analysis was conducted for each PCR to confirm the specificity of amplification. The expression level of the mtDNA and mitochondrial NADH dehydrogenase 1 (ND1) was normalized to *β*-actin and calculated as 2^−*ΔΔ*CT^. The primers are shown in Supplementary [Supplementary-material supplementary-material-1].

### 2.16. Measurement of the Liver ATP Content

The content of liver ATP was measured using an ATP assay kit according to the manufacturer's instructions. Briefly, after surgery, the liver tissues were washed with ice-cold PBS and then homogenized and sonicated in lysis buffer on ice. After sonication, the lysed tissues were centrifuged at 12000 g for 5 min to remove debris. Then, ATP was determined using an ATP assay kit based on the luciferin/luciferase assay and normalized for protein content.

### 2.17. Liver Hematoxylin and Eosin (HE) and Oil Red O Staining

Formalin-fixed liver specimens were embedded in paraffin blocks and cut into 5 *μ*m sections. In addition, liver biopsies were read and NAFLD activity (NAS) was scored using the NASH Clinical Research Network Histologic Scoring System [23]. The sections were then stained with HE for histology as described previously [22]. Fresh frozen liver sections were used to determine hepatic lipid content by staining with Oil Red O. The Oil Red O working solution was prepared as described above. Fresh frozen sections were air-dried at room temperature for 2 h and fixed with 4% paraformaldehyde for 5 min. The sections were washed with tap water for 10 min and then rinsed in 60% isopropanol to remove excess water. The sections were stained with freshly prepared Oil Red O working solution for 20 min and then rinsed in 60% isopropanol to remove excess Oil Red O. Nuclei were stained with hematoxylin for 2 min and then rinsed with tap water.

### 2.18. Immunohistochemistry (IHC)

For IHC, the sections were deparaffinized and rehydrated with ethanol and xylene and then heated to 95°C for 20 min in 10 mM citrate buffer, pH 6.0. After a blocking step with PBS containing 10% goat serum for 1 h at room temperature, the sections were incubated with a primary antibody 1 : 200 RNLS overnight at 4°C. Subsequently, the sections were incubated with biotinylated goat anti-rabbit IgG. After incubation with secondary antibodies, the sections were mounted and evaluated with an Olympus microscope. For the quantitative expression of RNLS, the density of the immunoreactive proteins was analyzed by Image Pro Plus software (Media Cybernetics Corporation, USA).

### 2.19. Terminal Deoxynucleotidyl Transferase dUTP Nick End Labeling (TUNEL) Staining

The TUNEL assay was performed according to the manufacturer's protocol. Briefly, sections were deparaffinized and then incubated with proteinase K for 15 min at room temperature. After incubating, sections were covered with TUNEL test solution, containing fluorescein-conjugated dUTP and TdT enzyme, at a ratio of 9 : 1 (*v*/*v*) in a humidified box at 37°C for 60 min. After washing, 4′,6-diamidino-2-phenylindole (DAPI) was added to stain the cell nuclei. The images were photographed by a fluorescent microscope (Olympus Corporation, Tokyo, Japan).

### 2.20. Immunofluorescence and Confocal Immunofluorescence

After treatment, the cells were fixed in 4% paraformaldehyde at room temperature for 30 min. Subsequently, the cells were incubated with 5% bovine serum albumin (BSA) for 1 h and then incubated with an anti-RNLS antibody (Abcam) or anti-TOM20 antibody (CST) at 4°C overnight. After 3 washes with PBS, the cells were incubated with goat anti-rabbit IgG (BA1105, BOSTER, or A0516, Beyotime) for 1 h. After washing, DAPI was added to stain the cell nuclei. Cell fluorescence was imaged on an epifluorescence microscope (Olympus Corporation, Tokyo, Japan) or on a confocal microscope (Zeiss Germany, Oberkochen, Germany). The RNLS fluorescence intensity was analyzed by ImageJ software.

### 2.21. Western Blot (WB) Analysis

Tissues or cell proteins were extracted using cold RIPA buffer containing PMSF. For the mitochondrial and cytoplasmic protein extraction, a cell mitochondria isolation kit was used to isolate mitochondria and cytosol using a previously described protocol [24]. The protein concentration was determined with a BCA protein assay kit. Total proteins (40 *μ*g) were loaded onto SDS-PAGE gels, separated by electrophoresis, and transferred onto PVDF membranes. After a blocking step in Tris-buffered saline (TBS) solution containing 5% nonfat milk and 0.1% Tween 20 for 1 h, the membranes were incubated with primary antibodies overnight at 4°C. After three washes with TBS solution containing 0.1% Tween 20, the membranes were incubated at 37°C with HRP-linked secondary antibody for 1 h. Enhanced chemiluminescent substrate was used to visualize the immune bands. ImageJ software was used to analyze the band density.

### 2.22. RNA Isolation, Reverse Transcription, and Quantitative Real-Time Polymerase Chain Reaction (qRT-PCR)

Total RNA from tissue samples or cells was extracted by using TRIzol reagent according to the manufacturer's instructions as previously described [25]. For qPCR, reverse transcription was conducted with HiScript® II Q RT SuperMix (+gDNA wiper) according to the manufacturer's protocol. Real-time PCR was performed as described above. The primers are shown in Supplementary [Supplementary-material supplementary-material-1].

### 2.23. Enzyme-Linked Immunosorbent Assay (ELISA)

The protein levels of RNLS in the serum were detected by using a mouse RNLS ELISA kit according to the manufacturer's instructions as previously described [15].

### 2.24. Microarray Data Information and Differentially Expressed (DE) Transcription Factor Identification

The gene expression profile of GSE89632 [26], containing 24 healthy control (HC) tissue samples, 20 simple steatosis (SS) tissue samples, and 19 nonalcoholic steatohepatitis (NASH) tissue samples, was obtained from Gene Expression Omnibus (GEO) (https://www.ncbi.nlm.nih.gov/geo), a free database of microarray profiles and next-generation sequencing. In addition, the GEO2R online tool (https://www.ncbi.nlm.nih.gov/geo/geo2r/) was applied to detect the DE genes (DEGs) in HC, SS, and NASH samples. *P* < 0.05 and ∣log fold change (FC)∣ ≥ 1 were set as cut-off criteria. For DE transcription factor identification, the Gene Transcription Regulation Database (GTRD) (http://gtrd.biouml.org/) was used to select downregulated DE transcription factors from DEGs. The heatmap of DE transcription factors was drawn by R software.

### 2.25. Statistical Analysis

**Figure 1:**
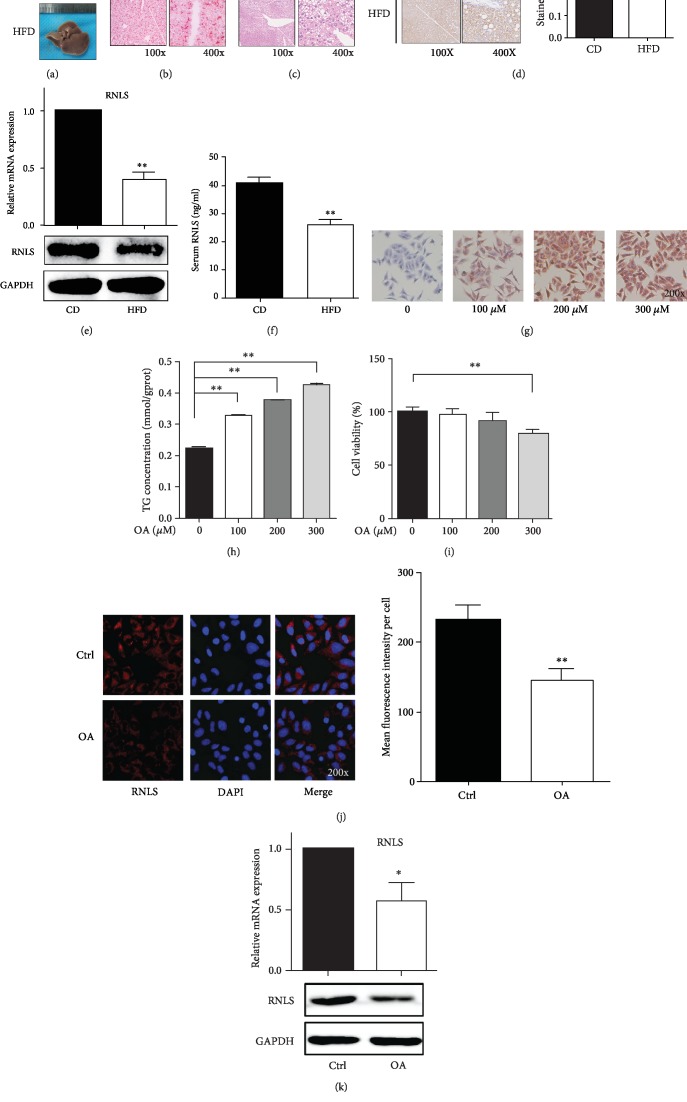
RNLS is downregulated in HFD-induced fatty livers and OA-induced steatotic HepG2 cells. (a) Gross liver from C57 mice fed a CD or a HFD for 20 weeks. Representative liver sections stained with HE (b) or Oil Red O (c) are shown. Original magnification, ×100 and ×400. (d) Immunohistochemical staining of RNLS in liver tissues from CD and HFD C57 mice. Original magnification, ×100 and ×400. The brown stained ratio in each group was analyzed. RNLS protein levels and relative mRNA expression in liver tissues and in the serum from CD and HFD C57 mice were evaluated by western blotting, RT-qPCR, and ELISA (e, f). (g) Oil Red O staining of HepG2 cells treated with different concentrations of OA. Original magnification, ×200. (h) TG concentration in HepG2 cells treated with different concentrations of OA. (i) Cell viability of HepG2 cells treated with different concentrations of OA. RNLS protein levels and relative mRNA expression in HepG2 cells treated with or without OA were evaluated by immunofluorescence (j), western blotting, and RT-qPCR (k). Original magnification, ×200. ^∗^*P* < 0.05, ^∗∗^*P* < 0.01. Data are plotted as the mean ± SD from three independent experiments. Abbreviation: CD: control diet; HFD: high-fat diet; OA: oleic acid; TG: triglyceride.

**Figure 2:**
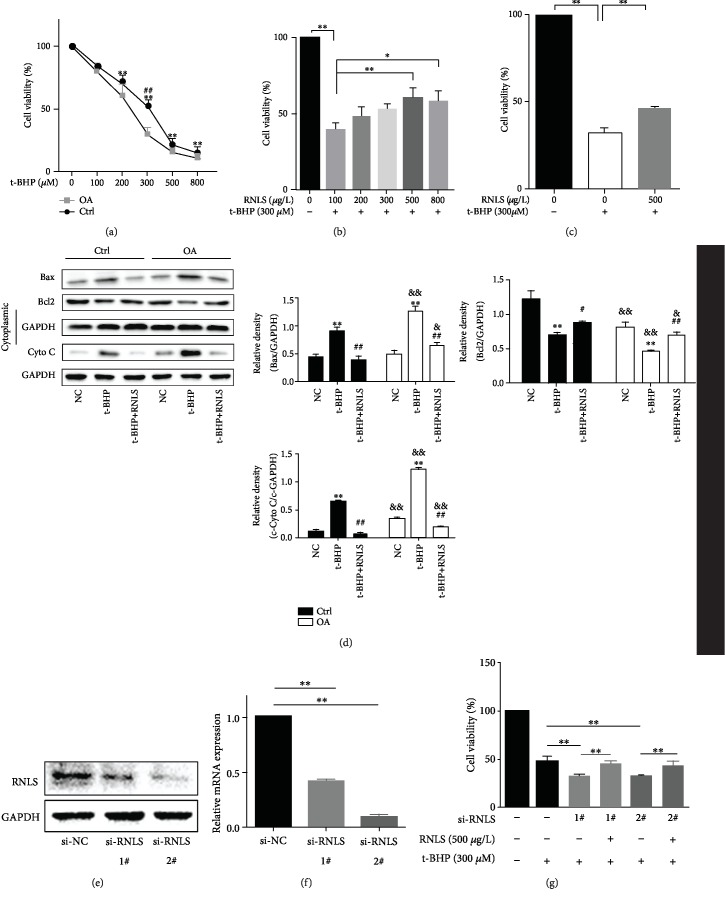
RNLS protects t-BHP-induced HepG2 cell oxidative stress injury in vitro. (a) Cell viability of HepG2 cells (Ctrl) and 200 *μ*M OA-induced steatotic HepG2 cells (OA) treated with t-BHP at different concentrations. ^∗^*P* < 0.05, ^∗∗^*P* < 0.01 t-BHP group vs. NC group; ^##^*P* < 0.01 Ctrl group vs. OA group. (b) Cell viability of HepG2 cells pretreated with RNLS at different concentrations, followed by 300 *μ*M t-BHP treatment. (c) Cell viability of OA-induced steatotic HepG2 cells pretreated with or without 500 *μ*M RNLS in an in vitro IR model induced by 300 *μ*M t-BHP treatment. (d) The expression levels of apoptosis-related proteins of hepG2 cells were evaluated by western blotting. ^∗∗^*P* < 0.01 vs. NC group, ^#^*P* < 0.05, ^##^*P* < 0.01 vs. t-BHP group; ^&^*P* < 0.05, ^&&^*P* < 0.01 vs. Ctrl group. (e, f) RNLS protein levels and relative mRNA expression in HepG2 cells transfected with si-RNLS were evaluated by western blotting and RT-qPCR. (g) Cell viability of RNLS-knockdown HepG2 cells pretreated with or without 500 *μ*g/L RNLS in an in vitro IR model. ^∗∗^*P* < 0.01. Data are plotted as the mean ± SD from three independent experiments.

**Figure 3:**
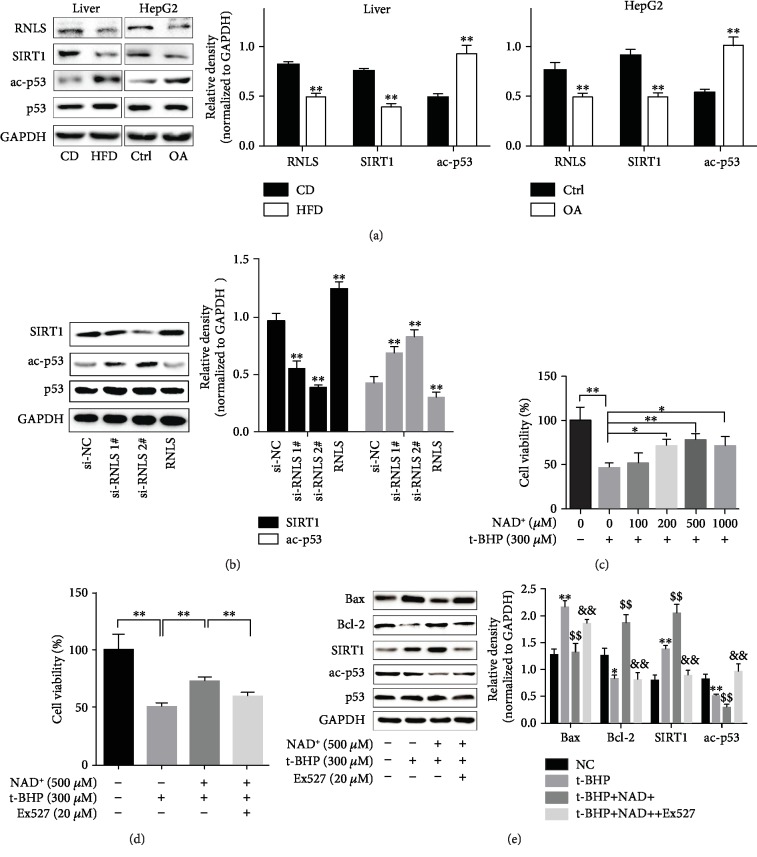
RNLS activates SIRT1 through increasing the NAD^+^ content to protect against liver IR injury. (a) RNLS, SIRT1 protein levels, and activity of SIRT1 in liver tissues and HepG2 cells. ^#^ac-p53 normalized to p53; ^∗∗^*P* < 0.01. (b) Expression and activity of SIRT1 in HepG2 cells with RNLS knockdown or RNLS administration. ^∗∗^*P* < 0.01 vs. si-NC group. (c) Cell viability of HepG2 cells pretreated with NAD^+^ at different concentrations followed by 300 *μ*M t-BHP treatment. (d) Cell viability of HepG2 cells pretreated with NAD^+^ followed by 300 *μ*M t-BHP treatment with or without EX527 administration. (e) Bax, Bcl-2, and SIRT1 protein levels and activity of SIRT1 in HepG2 cells pretreated with NAD^+^ followed by 300 *μ*M t-BHP treatment with or without EX527 administration. ^#^ac-p53 normalized to p53; ^∗^*P* < 0.05, ^∗∗^*P* < 0.01 vs. NC group; ^$$^*P* < 0.01 vs. t-BHP group, ^&&^*P* < 0.01 vs. t-BHP+NAD^+^ group. Data are plotted as the mean ± SD from three independent experiments.

**Figure 4:**
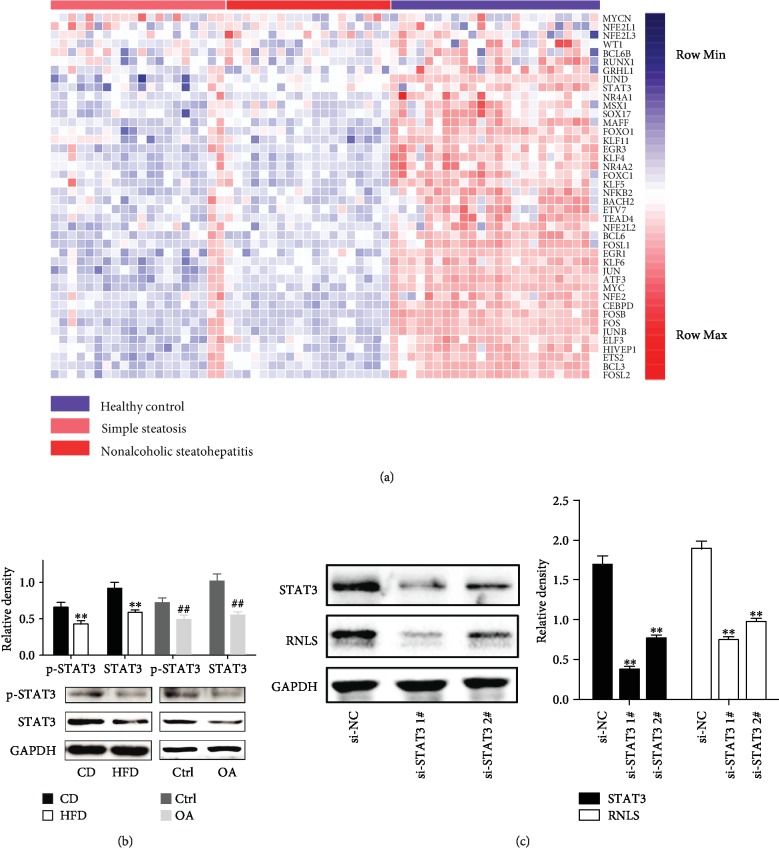
RNLS is transcriptionally downregulated by the decrease of STAT3 under HFD conditions. (a) Heatmap plot of differentially expressed genes among livers from the healthy control, simple steatosis, and nonalcoholic steatohepatitis. (b) The expression and phosphorylation levels of STAT3 protein in mice and HepG2 cells. ^∗∗^*P* < 0.01 vs. CD group, ^##^*P* < 0.01 vs. Ctrl group. (c) The expression levels of STAT3 and RNLS in STAT3 knockdown cells. ^∗∗^*P* < 0.01 vs. si-NC group. Data are plotted as the mean ± SD from three independent experiments.

**Figure 5:**
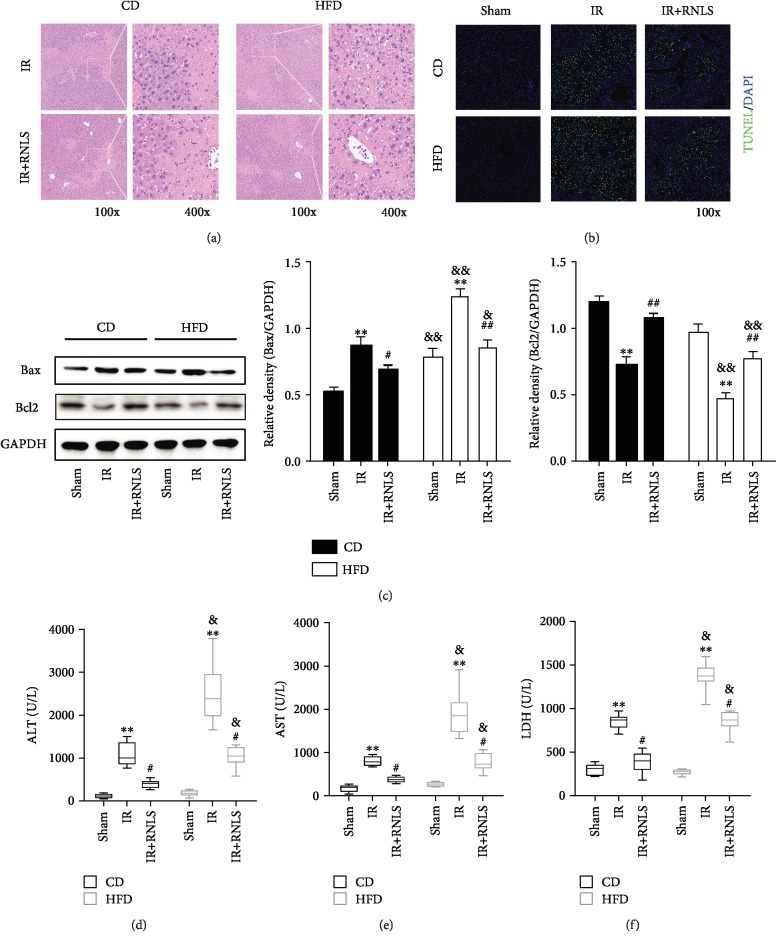
RNLS protects against IR-induced hepatic necrosis in vivo. (a) The livers of the IR and IR+RNLS groups from CD and HFD mice were subjected to histological evaluation by HE staining. Original magnification, ×100 and ×400. (b) TUNEL staining of the livers of the sham, IR, and IR+RNLS groups from CD and HFD mice. Original magnification, ×100. (c) The expression levels of liver apoptosis related proteins were evaluated by western blotting. (d–f) Liver enzymes ALT, AST, and LDH were determined in the serum of sham, IR, and IR+RNLS mice from both CD and HFD groups. ^∗∗^*P* < 0.01 vs. sham group, ^#^*P* < 0.05, ^#^*P* < 0.05, and ^##^*P* < 0.01 vs. IR group; ^&^*P* < 0.05, ^&&^*P* < 0.01 vs. CD group. Data are plotted as the mean ± SD from three independent experiments. Box plots: horizontal line within the box represents the median value, the upper and the lower limits of box indicate the 25th and 75th percentiles, and the capped vertical lines represent the minimum and maximum values. Abbreviation: TUNEL: terminal deoxynucleotidyl transferase dUTP nick end labeling; DAPI: 4′,6-diamidino-2-phenylindole.

The experiment data were analyzed by Statistical Package for Social Sciences (SPSS version 22.0; IBM Analytics, Chicago, IL, USA) and GraphPad Prism 6.0 statistical software (GraphPad Software, Inc., La Jolla, CA, USA). All data were presented as the mean values ± standard deviation (SD) for normal data and median ± interquartile range for nonnormal data. Statistical significance was determined with unpaired two-tailed Student's *t*-test between two groups. For multiple groups, significance was evaluated by one-way analyses of variance (ANOVA), followed by Tukey's test or Dunnett's test (Figures 1(h), 1(i), 2(b), 2(f), 3(b), 3(c), and 4(c), Supplementary Figures [Supplementary-material supplementary-material-1], [Supplementary-material supplementary-material-1], [Supplementary-material supplementary-material-1], and [Supplementary-material supplementary-material-1]) or two-way ANOVA followed by Tukey's test. Statistical significance of differing variables was determined via the nonparametric Kruskal-Wallis test followed by Dunn's test (Figures 5(d)–5(f)). All experiments and assays were independently repeated at least three times. When *P* < 0.05, the values were considered statistically significant.

## 3. Results

### 3.1. RNLS Is Downregulated in Liver and Hepatocellular Steatosis and Elevated in Liver IR and Hepatocellular Oxidative Stress

To investigate whether RNLS is involved in hepatic steatosis, we examined hepatic RNLS expression in C57BL/6 mice fed a HFD. As shown in Table 1, the body weight, liver TG and TC content, and NAFLD activity score (NAS) were higher in HFD mice than CD mice. Besides, as shown in Figure 1(a), after 20-week HFD feeding, the mouse liver color changed from red to ashen, displaying gross liver steatosis. The steatosis was further confirmed by histological sections with Oil Red O and HE staining, which showed hepatic lipid accumulation (Figures 1(b) and 1(c)). The protein and mRNA levels of RNLS were detected by IHC, WB, and qRT-PCR, which showed a significant reduction in the steatotic liver (Figures 1(d) and 1(e)). Since RNLS is a secretory protein, we further examined the serum level of RNLS. Consistently, the serum level of RNLS was also decreased in HFD mice (Figure 1(f)).

HepG2 cultured in OA medium were used to induce hepatic steatosis in vitro. As shown in Figures 1(g) and 1(h), at biologically safe concentrations of DMSO (Supplementary [Supplementary-material supplementary-material-1]), with the increasing of OA concentration, the lipid accumulation and TG level in HepG2 cells were markedly increased. Additionally, the 100 and 200 *μ*M OA exerted no cytotoxicity (Figure 1(i)); thus, 200 *μ*M OA was chosen for inducing hepatic steatosis in vitro. Moreover, the protein and mRNA levels of RNLS were detected by IF, WB, and qRT-PCR, which showed a significant reduction in hepatic steatosis (Figures 1(j) and 1(k)). These results indicated the possible involvement of RNLS in hepatic steatosis.

**Figure 6:**
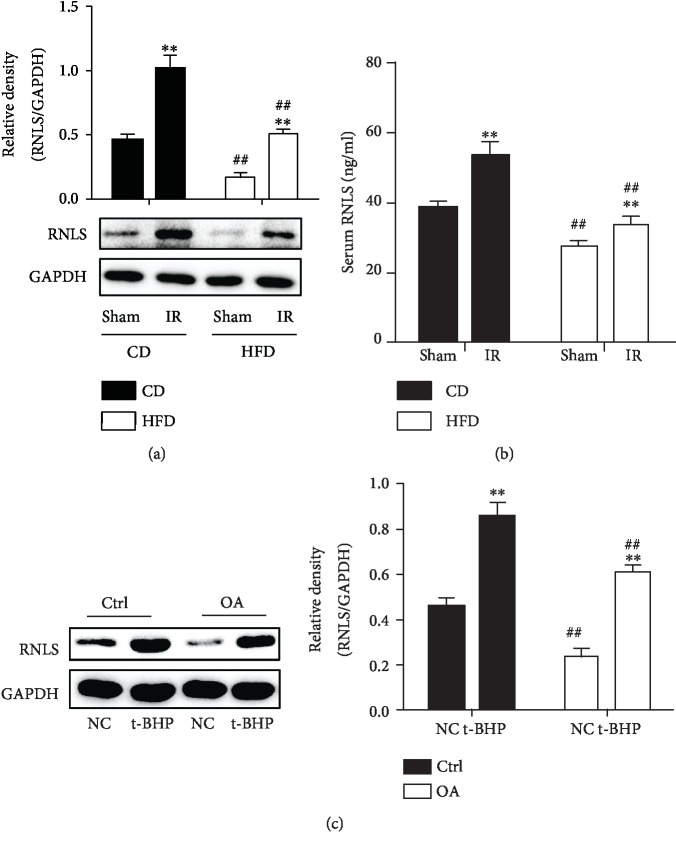
RNLS is elevated in liver IR and hepatocellular oxidative stress. RNLS protein levels in liver tissues and in the serum from CD and HFD C57 mice exposed to liver IR were evaluated by western blotting and ELISA (a, b). RNLS protein levels in Ctrl and OA HepG2 cells under t-BHP treatment were evaluated by western blotting (c). ^∗∗^*P* < 0.01 vs. sham or NC group, ^##^*P* < 0.01 vs. CD or Ctrl group. Data are plotted as the mean ± SD from three independent experiments.

As shown in Figures 6(a) and 6(b), after 90 min of ischemia and 2 h of reperfusion, the expression of mice liver RNLS and serum RNLS concentration were elevated significantly in both CD and HFD groups. Besides, the RNLS levels in CD groups were lower than that in HFD groups. In addition, in in vitro experiments, RNLS also increased markedly after 300 *μ*M t-BHP treatment in both Ctrl and OA groups, and the RNLS levels in OA groups were lower than that in Ctrl groups. These results further indicated the possible involvement of RNLS in hepatic IR injury.

### 3.2. Fatty Livers Are More Susceptible to IR Injury In Vivo and In Vitro

As shown in Figures 5(a)–5(c), IR induced marked necrosis and apoptosis in liver tissues. In addition, the necrotic area and apoptosis were significantly larger in the HFD group than that in the CD group. Consistent with the effects of necrosis, the serum levels of ALT, AST, and LDH were significantly elevated after IR. Moreover, the levels of these liver injury markers in the HFD group were significantly higher than those in the CD group (Figures 5(d)–5(f)).

Additionally, RNLS administration alone did not alter the TC and TG content, necrosis, and apoptosis of the liver in both CD and HFD groups (Supplementary Figures [Supplementary-material supplementary-material-1], [Supplementary-material supplementary-material-1], [Supplementary-material supplementary-material-1], and [Supplementary-material supplementary-material-1]). Besides, there was no difference between the right lobe of liver in IR group mice, which was not suffered from ischemia and subsequent restoration of blood flow, and sham group mice in necrosis, apoptosis, and liver enzymes (Supplementary Figures [Supplementary-material supplementary-material-1]–[Supplementary-material supplementary-material-1]).

The in vitro hepatocyte oxidative stress model exhibited worse cell viability in the OA groups than in the Ctrl groups, after 3 h of 300 *μ*M t-BHP treatment (Figure 2(a)). 300 *μ*M t-BHP was thus chosen for inducing oxidative stress in vitro. Additionally, t-BHP treatment promoted the activation of cell apoptosis through increasing the release of mitochondrial cytochrome C (cyto C), upregulating the expression of Bax, and downregulating the expression of Bcl-2. The activation of apoptosis was higher in OA-induced steatotic cells.

### 3.3. RNLS Protects against Liver IR Injury In Vivo and In Vitro

Compared to the CD-IR and HFD-IR groups, RNLS supplementation effectively alleviated the necrotic size and apoptosis of liver tissues (Figures 5(a)–5(c)) and decreased the serum levels of ALT, AST, and LDH (Figures 5(d)–5(f)).

In the in vitro hepatocyte oxidative stress model, supplementation with exogenous RNLS at a concentration of 500-800 *μ*M improved cell viability in hepatocytes under oxidative stress after t-BHP treatment. In addition, 500 *μ*M RNLS showed the best protective effect against oxidative stress; thus, 500 *μ*M RNLS was chosen for further experiments (Figure 2(b)). Moreover, RNLS supplementation also improved the cell viability of steatotic hepatocytes under oxidative stress (Figure 2(c)). Besides, RNLS administration could effectively attenuate cell apoptosis by suppressing cyto C liberation and Bax expression and elevating Bcl-2 expression (Figure 2(d)).

To confirm the protective effect of RNLS in vitro, HepG2 cells were stably transfected with si-RNLS and then exposed to t-BHP. The protein and mRNA levels of RNLS were decreased significantly compared to those of si-NC cells (Figures 2(e) and 2(f)). As shown in Figure 2(g), the depletion of RNLS leads to worse cell viability than that of si-NC cells under t-BHP treatment, and RNLS supplementation effectively restored cell viability.

### 3.4. RNLS Suppresses IR- and t-BHP-Induced Oxidative Stress Level In Vivo and In Vitro

**Figure 7:**
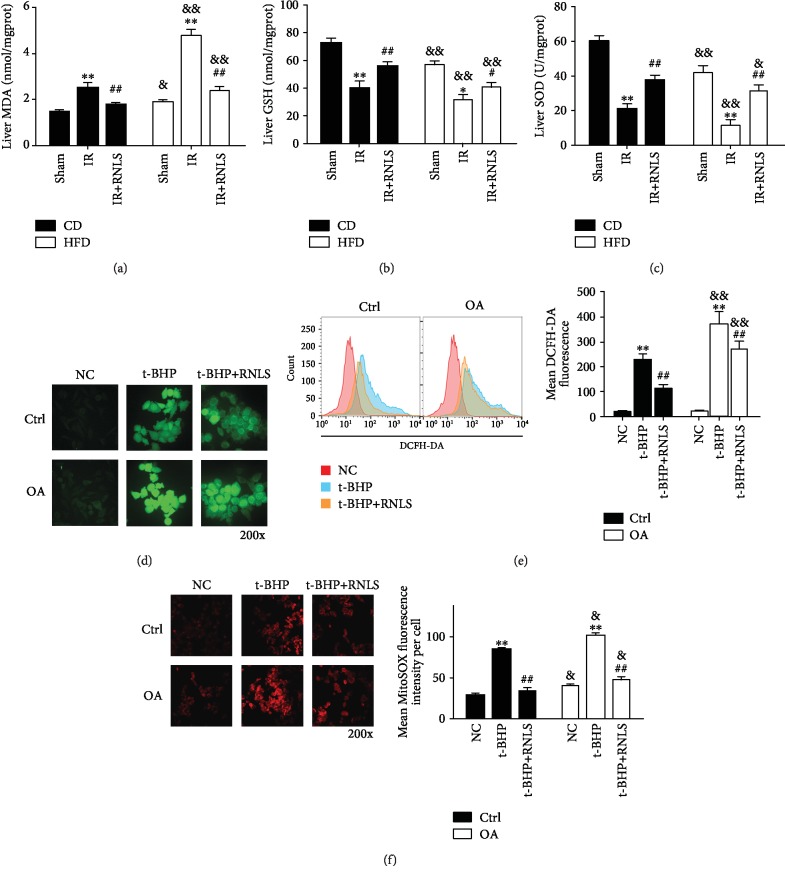
RNLS suppresses IR- and t-BHP-induced oxidative stress in vivo and in vitro. (a–c) MDA and GSH levels and SOD activity in the liver tissues of sham, IR, and IR+RNLS from both CD and HFD mice. (d, e) Intracellular ROS was determined by DCFH-DA staining using fluorescence microscopy and flow cytometry. (f) Mitochondrial ROS was detected by MitoSOX red mitochondrial superoxide indicator using fluorescence microscopy. Original magnification, ×200. ^∗∗^*P* < 0.01 vs. sham or NC group; ^#^*P* < 0.05, ^##^*P* < 0.01 vs. IR or t-BHP; ^&^*P* < 0.05, ^&&^*P* < 0.01 vs. CD or Ctrl group. Data are plotted as the mean ± SD from three independent experiments. Abbreviation: MDA: malondialdehyde; GSH: glutathione; SOD: superoxide dismutase; DCFH-DA: 2′,7′-dichlorofluorescin diacetate.

Since oxidative stress is the main mechanism involved in liver IR injury, we next investigated the effect of RNLS on mediating oxidative stress. The concentration of MDA and GSH and the activity of SOD in liver tissues were determined. Moreover, the intracellular and mitochondrial ROS content were also evaluated. As shown in Figures 7(a)–7(c), liver IR induced significant increases in the MDA level and reduced the GSH content and SOD activity in both CD and HFD mouse livers. Moreover, RNLS supplementation alleviated the effect on the MDA level and restored the liver antioxidative capacity through upregulating GSH content and SOD activity. Consistently, in vitro experiments showed that t-BHP treatment significantly elevated the intracellular ROS content in both Ctrl and OA groups, and RNLS administration suppressed the intracellular ROS levels in these two groups (Figures 7(d) and 5(e)). Additionally, HFD alone significantly elevated the tissue MDA level and reduced GSH content and SOD activity, and steatotic liver and HepG2 cells displayed higher levels of oxidative stress under IR or t-BHP treatment.

Since mitochondria are the major sources of ROS, we further detected the levels of mitochondrial ROS. As shown in Figure 7(f) t-BHP treatment significantly elevated the mitochondrial ROS content in both Ctrl and OA groups, and RNLS administration suppressed the mitochondrial ROS levels in these two groups, indicating that the elevated cellular oxidative stress may due to the mitochondrial ROS production.

### 3.5. RNLS Alleviates the IR- and t-BHP-Induced Mitochondrial Dysfunction

**Figure 8:**
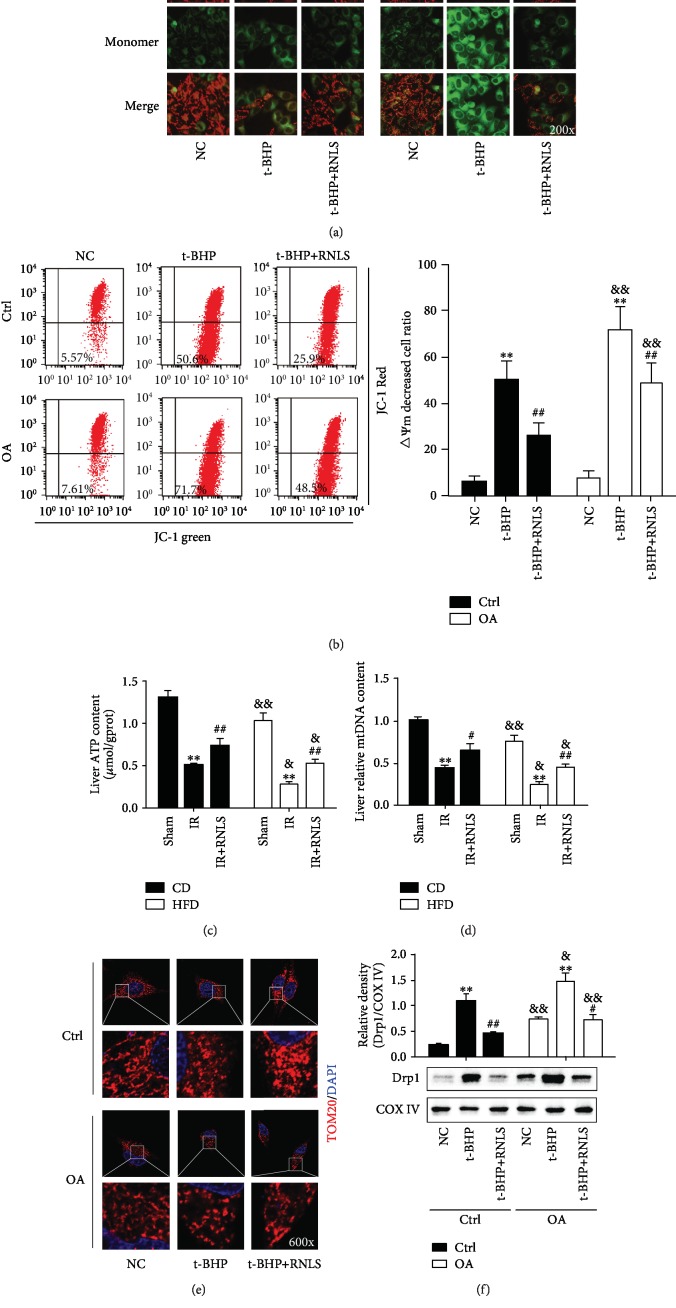
RNLS alleviates the IR- and t-BHP-induced mitochondrial dysfunction. Mitochondrial membrane potential (*ΔΨ*m) was determined by JC-1 staining using fluorescence microscopy (a) and flow cytometry (b). Liver ATP content (c) and mtDNA copy numbers (d). Representative images showing mitochondrial morphology visualized by TOM20 staining (red) using confocal microscopy; DAPI-stained nuclei were blue (e). Original magnification, ×600. The levels of mitochondrial Drp1 in each group were determined by western blotting (f). ^∗∗^*P* < 0.01 vs. NC or sham group; ^#^*P* < 0.05, ^##^*P* < 0.01 vs. t-BHP or IR group; ^&^*P* < 0.05, ^&&^*P* < 0.01 vs. Ctrl or CD group. Data are plotted as the mean ± SD from three independent experiments.

Since mitochondria are also the first target of ROS, we next investigated the effect of RNLS on the preservation of mitochondrial function (ATP production, mtDNA copy numbers, and mitochondrial dynamics) in liver IR injury. As shown in Figure 8(a), the cells were stained with less red fluorescence (representing high *ΔΨ*m mitochondria) and more green fluorescence (representing low *ΔΨ*m mitochondria) in the t-BHP treatment groups than in the NC groups, indicating a significant loss of *ΔΨ*m after t-BHP treatment. RNLS pretreatment restores cell *ΔΨ*m. In addition, cells in the OA group displayed a significantly lower *ΔΨ*m level than those in the Ctrl group with or without RNLS supplementation under t-BHP treatment. Additionally, the same results were shown using a flow cytometry method (Figure 8(b)). These data indicated that steatotic hepatocytes are susceptible to t-BHP-induced *ΔΨ*m collapse, and RNLS could ameliorate liver IR injury via preventing *ΔΨ*m collapse.

In addition, in in vivo experiments, liver ATP content and mtDNA copy numbers were decreased markedly in IR, which increased by RNLS administration. Besides, HFD mouse livers presented lower ATP content and mtDNA copy numbers than those in CD mice (Figures 8(c) and 8(d)).

Previous studies have shown that IR promotes mitochondrial fission, induces mitochondrial fragmentation, and represses mitochondrial fission-attenuated IR-induced cell death [27, 28]. These findings highlighted mitochondrial fission as a negative regulator of mitochondrial homeostasis and a therapeutic target for IR injury.

To explore the beneficial effects of RNLS on mitochondrial homeostasis, we investigated the alteration of mitochondrial fission. As shown in Figure 8(e), t-BHP treatment significantly induced mitochondrial fragmentation in both Ctrl and OA groups, and RNLS administration restored the mitochondrial network morphology. Consistently, the mitochondrial translocation of the mitochondrial fission-related protein Drp1 was elevated after t-BHP treatment, and RNLS administration suppressed the alteration in the proteins (Figure 8(f)). Additionally, OA treatment alone also induced mitochondrial fission and promoted Drp1 mitochondrial translocation, and under t-BHP-induced oxidative stress, the OA group displayed more severe mitochondrial fragmentation and higher Drp1 expression than did the Ctrl group, with or without RNLS administration. In summary, these data intensively indicated that RNLS protected liver IR injury through improving mitochondrial function.

### 3.6. RNLS Activates SIRT1 to Protect against Liver IR Injury

We found that the expression and activity of not only RNLS but also SIRT1 were downregulated in steatotic liver and hepatocytes (Figure 3(a)). We further investigated whether RNLS could mediate the expression and activity of SIRT1. As shown in Figure 3(b), RNLS knockdown leads to the significant downregulation of SIRT1 expression and activity, and RNLS administration upregulated the expression and activity of SIRT1. We then explored the mechanism of how RNLS regulates SIRT1.

NAD^+^ is an indispensable substrate for SIRT1, which exerts a variety of cytoprotective activities. Previous studies have shown that RNLS specifically oxidizes *α*-NADH and converts it into *β*-NAD^+^ [16]. In addition, the depletion of RNLS significantly downregulated NAD^+^ levels [29–31]. These data indicated that RNLS may activate SIRT1 though elevating NAD^+^ levels. To further investigate whether NAD^+^ is involved in the protective effect of RNLS against liver IR injury, the cells were pretreated with biologically safe concentrations of NAD^+^ (100-1000 *μ*M) at 1 h before t-BHP treatment (Supplementary [Supplementary-material supplementary-material-1]). As shown in Figure 3(c), 200-1000 *μ*M NAD^+^ significantly improved cell viability, and 500 *μ*M NAD^+^ exerted the greatest protective effect. Additionally, 500 *μ*M NAD^+^ supplementation also improved steatotic hepatocyte viability after t-BHP treatment (Supplementary [Supplementary-material supplementary-material-1]). Additionally, we further investigated whether the cytoprotection of NAD^+^ supplementation was due to the promotion of SIRT1 activity. We blocked SIRT1 activity using biologically safe concentrations of EX527 (0-20 *μ*M) (Supplementary [Supplementary-material supplementary-material-1]) to observe changes in the effects of NAD^+^ supplementation on cell viability, p53 acetylation, and Bcl-2 and Bax expression in t-BHP-treated cells. As shown in Figure 3(d) and Supplementary [Supplementary-material supplementary-material-1], EX527 administration partially abolished the protective effect of NAD^+^ supplementation on cell viability under t-BHP treatment. Moreover, NAD^+^ markedly decreased the acetylation level of p53, negatively regulated the expression of Bax, and increased the expression of Bcl-2. However, EX527 administration inhibited the activity of SIRT1, blocked the effects of NAD^+^ supplementation, increased the acetylation level of p53, positively regulated the expression of Bax, and decreased the expression of Bcl-2 (Figure 3(e)).

### 3.7. RNLS Is Transcriptionally Downregulated by the Decrease in STAT3 Expression under HFD Conditions

To further investigate the mechanism underlying the downregulation of RNLS in fatty livers, we downloaded a microarray dataset containing normal and fatty liver samples from the GEO database. Bioinformatic analysis was then used to identify DE transcription factors among the different groups. After the analysis, 43 downregulated DE transcription factors were identified. In addition, we found that STAT3 was significantly downregulated in fatty livers (Figure 4(a)). In addition, our WB (Figure 4(b)) and qRT-PCR (Supplementary Figures [Supplementary-material supplementary-material-1] and [Supplementary-material supplementary-material-1]) results in mouse liver tissues and HepG2 cells further showed that the expression and phosphorylation of STAT3 were deceased in steatotic livers and cells (Figure 4(b)). Additionally, a recent study showed that RNLS is a direct transcriptional target of STAT3 [32]. We also found that downregulation of STAT3 decreased the expression of RNLS markedly (Figure 4(c)). In summary, these data intensively indicated that RNLS is transcriptionally downregulated by the decrease in STAT3 expression under HFD conditions.

## 4. Discussion

Liver IR injury, which may occur in liver resections and transplantations, traumas, and vascular surgeries, is a serious complication in the clinical practice. The excessive production of ROS during IR and subsequent disorder of redox balance are the most invoked mechanisms in liver IR injury. The liver is particularly prone to ROS production due to its high metabolic rate and because hepatocytes are rich in ROS-producing mitochondria, cytochrome P450 enzymes, and iNOS [5]. Moreover, IR injury, which in most severe cases culminates in acute liver failure, is particularly pronounced in livers affected by NAFLD. Operative mortality associated with steatosis exceeds 14%, compared with 2% mortality observed for healthy livers, and the risks of primary nonfunction and dysfunction after surgery are also higher [33]. NAFLD development is a “two-hit” process. The “first hit” is the lipid accumulation in hepatocytes, and the “second hit” consists of inflammation, cell death, extracellular matrix remodeling by hepatic stellate cells, and fibrosis [34]. NAFLD is characterized by fat accumulation in hepatocytes, which provides a potential substrate for lipid peroxidation and ROS toxicity. Excessive ROS production enhances lipid peroxidation, which subsequently leads to the formation of other reactive metabolites in the liver, such as MDA [35]. Additionally, accumulating evidence indicates that mitochondrial dysfunction is involved in the physiopathology of NAFLD. Patients with nonalcoholic steatohepatitis (NASH) show mitochondrial morphology changes and dysfunction, including the presence of paracrystalline inclusions, decreased liver mitochondrial DNA levels, and reduced activity of complexes I, III, IV, and V, thereby decreasing ATP synthesis [36]. The possible mechanisms may involve lipid peroxidation, TNF-*α* expression, and ROS production [36]. Moreover, recent studies have shown that HFD promotes Drp1 translocation to mitochondria, forcing mitochondrial fission. The excessive fission dramatically mediated mitochondrial dysfunction, including extensive mPTP opening, reduced *ΔΨ*m, oxidative stress, calcium overload, mitochondrial respiratory collapse, and ATP shortage [37]. Consistent with previous findings, our present results showed that HFD treatment significantly increased MDA levels and lowered GSH level and SOD activity. Additionally, OA treatment enhanced mitochondrial fission in hepatocytes. These results indicated that a higher oxidative stress level and impaired mitochondrial function might be attributed to the susceptibility of livers with NAFLD to IR injury.

Suppressing ROS burst in oxidative stress is an efficient way to alleviate liver IR injury. Antioxidant strategies, including ischemia preconditioning (IPC) [38], and antioxidant pharmacological therapies, including N-acetylcysteine [39], *α*-tocopherol [40], *β*-carotene [41], vitamin C [42], *trans*-resveratrol [43], tetrandrine [44], and extracts from green tea [45], grape seeds [46], and other plants, confer protection from liver IR injury by attenuating oxidative stress and maintaining the cellular redox balance. Catecholamines, including dopamine, are subjected to autoxidation and produce ROS [47], which is implicated in IR injury [48, 49]. RNLS is widely defined to be an enzyme whose function is to oxidize circulating catecholamines [48], indicating a potential antioxidant role of RNLS. Consistent with previous findings, our results showed that RNLS exerted a potent antioxidant property that attenuated t-BHP-induced cellular and mitochondrial ROS production, reduced IR-induced liver MDA augmentation, and increased liver SOD activity and GSH levels.

Mitochondria are the main sources and the first targets of ROS during IR injury. The mitochondrial electron transport chain (ETC) is the most important source of ATP in mammalian cells. Nevertheless, during electron transfer, 1-3% of electrons escape to generate superoxide radicals instead of reducing oxygen to water [50]. Several redox centers (complexes I and III) of the mitochondrial ETC leak electrons to molecular oxygen and thus play chief roles in superoxide production [51]. As mitochondria are the major generators of ROS, this organelle often becomes the target of elevated ROS exposure with fatal consequences because the ROS reaction site and formation site are close. Elevated levels of O_2_^−^ and HO^−^ associated with mtDNA damage are considered to play a major role in cell apoptosis [52]. Additionally, H_2_O_2_ directly oxidizes the mitochondrial membrane lipid and mitochondrial proteins, modifying their structural and functional properties and resulting in mitochondrial damage [53]. Besides, under oxidative stress conditions, the production of ROS in mitochondrial ETC is a self-enhancement process, which has been termed “ROS-induced ROS release” (RIRR) [9]. Damaged mitochondria show decreased *ΔΨ*m and cannot produce sufficient ATP, leading to cell edema. Moreover, damaged mitochondria generate excessive ROS and liberate proapoptotic factors into the cytoplasm, initiating cell apoptosis. Consistent with the above finding, our study showed that IR induced decreasing of cellular ATP content and mtDNA copy numbers. t-BHP-induced oxidative stress leads to a significant reduction in *ΔΨ*m and subsequent cell apoptosis. Moreover, RNLS administration preserved cellular ATP content, mtDNA copy numbers, and *ΔΨ*m and suppressed cell apoptosis. Accordingly, we could conclude that the progression of liver IR injury seems to depend on mitochondrial dysfunction.

Apart from ROS production, recent studies have reported that structural changes in mitochondria might also be relevant to IR-induced cell death [27, 28]. During liver IR, mitochondria affected by perturbations of the intra- or extracellular microenvironment activate protective systems to maintain homeostasis. Mitochondrial dynamics is one of the regulators of mitochondrial homeostasis, which are essential for cell viability and apoptosis and for mediating bioenergetic adaptation [54]. Mitochondria continually adapt their shapes through fusion, fission, and cristae remodeling in response to changes in energy demand and supply, and any impairment in this dynamic behavior could lead to cellular dysfunction or death [54]. Mitochondrial fission, a division event that produces one or more daughter mitochondria, occurs when mitochondria are damaged and subjected to high levels of cellular stress and cell death [55], which is regulated by the activation and subsequent mitochondrial recruitment of dynamin-related protein 1 (Drp1) [55]. In addition to the specific function in maintaining cellular homeostasis, excessive mitochondrial fission and fragmentation were found during apoptosis [56]. Besides, the inhibition of Drp1 suppresses the conversion of the mitochondrial phenotype to fragmentation and prevents the loss of *ΔΨ*m, the release of cytochrome C, and activation of caspase 3. Collectively, the inhibition of Drp1 blocks cell death, implicating mitochondrial fission as an important step in apoptosis [57]. In line with this finding, mitochondrial fission also plays a vital role in mediating cell death under IR injury [27]. Previous studies have shown that mitochondrial fission and fragmentation were markedly promoted in an IR- or H_2_O_2_-induced oxidative stress model and repressed mitochondrial fission through inhibiting Drp1 activity-attenuated IR-induced cell death [27, 28, 58–60]. Consistent with previous findings, our results showed that t-BHP-induced oxidative stress promoted mitochondria fission and cell apoptosis. Moreover, RNLS administration attenuated mitochondrial fission and reduced ROS-induced cell apoptosis.

SIRT1, a class III histone deacetylase whose activity is dependent on NAD^+^, regulates a wide array of cellular processes, including oxidative stress, metabolism, apoptosis, and aging by deacetylating both histone and nonhistone proteins [61]. SIRT1 plays a protective role against IR injury in the heart [61], kidney [62], brain [63], and liver [64]. Previous studies have demonstrated that SIRT1 deacetylates its downstream targets, including NF-*κ*B, Foxos, p53, and peroxisome proliferator-activated receptor gamma coactivator-1*α* (PGC-1*α*), to regulate apoptosis, inflammation, and oxidative stress and protect organs from IR injury [65]. Additionally, recent studies have shown that the SIRT1/PGC-1*α* [66] and SIRT1/p53 [67] pathways inhibited Drp1-mediated mitochondrial fission and subsequently induced mitochondrial dysfunction and mitochondria-derived ROS production, resulting in the attenuation of cell apoptosis and death. Consistent with previous studies, our results showed that RNLS administration significantly increased the activity of SIRT1 and protected against oxidative stress injury induced by t-BHP.

NAFLD is a clinicopathologic syndrome that encompasses several clinical entities ranging from simple steatosis to steatohepatitis, fibrosis, and end-stage liver disease. A variety of chemicals, mainly drugs, and diets have been implicated in hepatic steatosis [68]. Moreover, recent studies have indicated that a number of genes play important roles in the development of NAFLD [69]. In the present study, we found that STAT3 was downregulated in NAFLD through bioinformatic analysis and further verified this result by RT-qPCR and western blotting. Additionally, a recent study showed that RNLS is a direct transcriptional target of STAT3 [32], indicating that RNLS is transcriptionally downregulated by the decrease of STAT3 under HFD conditions. Furthermore, increasing evidence has shown that STAT3 protects against IR injury in multiple organs [70–72], indicating that RNLS may participate in the protective effect of STAT3 against IR injury. However, the role of STAT3 in the development of NAFLD remains controversial. A recent study identified p-STAT3 as a potential marker of histologic severity in hepatic steatosis, which may play a role in mechanisms of disease progression in patients with liver steatosis [73]. In contrast, previous studies have shown that activating the IL6/STAT3 signaling pathway markedly ameliorates liver lipid metabolism, inflammation, and insulin resistance under HFD [74, 75]. Thus, further studies are needed to elucidate the role of STAT3 in the progression of NAFLD and to investigate the mechanisms of the decline in STAT3 expression in NAFLD.

However, there are limitations in our present study. The first one is the adoption of liver cancer cell line, HepG2, which may be different from primary hepatocytes in response to OA or t-BHP treatment. Besides, oxidative stress induced by t-BHP may not completely mimic the pathophysiological process of liver IR injury.

## 5. Conclusions

To our knowledge, this study is the first report to demonstrate the downregulation of RNLS in NAFLD and the protective role of RNLS against liver IR injury, indicating that the downregulation of RNLS may be attributed to the susceptibility of the fatty liver to IR injury. Moreover, our findings have revealed that the mechanism of the protective role of RNLS in antioxidation and improving mitochondrial function involves the upregulation of NAD^+^ levels and the subsequent activation of SIRT1. Furthermore, the downregulation of RNLS in NAFLD is transcriptionally mediated by STAT3. Finally, our findings provided the basis for a novel therapeutic strategy for enhancing hepatocyte survival in patients, particularly NAFLD patients who suffer from liver IR. However, it is far away from clinical application; numbers of researches are yet to confirm its role in large animal experiments and reveal the molecular mechanisms.

## Figures and Tables

**Table 1:** Fundamental characteristics of mice in the study.

Items	Groups					
	CD (*n* = 15)			HFD (*n* = 15)		
	Sham	IR	IR+RNLS	Sham	IR	IR+RNLS
Age (week)	28	28	28	28	28	28
Body weight (g)	27.3 ± 0.76	27.6 ± 0.55	28.0 ± 0.35	37.0 ± 1.52^∗^	35.9 ± 0.31^∗^	37.0 ± 0.48^∗^
Liver weight (g)	1.16 ± 0.02	1.24 ± 0.04	1.20 ± 0.03	1.34 ± 0.04^∗^	1.18 ± 0.02	1.26 ± 0.05
HIS (%)	4.25 ± 0.09	4.48 ± 0.09	4.28 ± 0.08	3.65 ± 0.20^∗^	3.34 ± 0.03^∗^	3.41 ± 0.13^∗^
NAS score	0.40 ± 0.24	NA	NA	3.20 ± 0.20^∗^	NA	NA
TG (mmol/gprot)	0.04 ± 0.01	0.03 ± 0.00	0.04 ± 0.01	0.10 ± 0.00^∗^	0.10 ± 0.02^∗^	0.11 ± 0.02^∗^
TC (mg/gprot)	4.55 ± 0.56	4.41 ± 0.61	4.49 ± 0372	9.17 ± 0.83^∗^	9.34 ± 0.49^∗^	9.51 ± 0.51^∗^

CD: control diet; HFD: high-fat diet; HIS: hepatosomatic indices ((liver weight/body weight) × 100%); NAS score: NAFLD activity score; NA: not available; TG: triglyceride; TC: total cholesterol (^∗^*P* < 0.05 vs. CD groups).

## Data Availability

The Tables and Figures used to support the findings of this study are available from the corresponding author upon request.

## Supplementary Materials

Supplementary Materials Supplementary Table 1: the sequences of PCR primers. Supplementary Table 2: the sequences of siRNAs. Supplementary Figure 1: the synthesis of recombinant RNLS and its role in the liver without exposing to IR. Supplementary Figure 2: the protective effect of NAD^+^ on oxidative stress in steatotic HepG2 cells.
