# Supplementary material for: Renalase Attenuates Mouse Fatty Liver Ischemia/Reperfusion Injury through Mitigating Oxidative Stress and Mitochondrial Damage via Activating SIRT1
Source: Oxid Med Cell Longev. 2019 Dec 14;2019:7534285. doi: 10.1155/2019/7534285 (PMC6948337; doi:10.1155/2019/7534285)
Supplement: Supplementary Materials — Supplementary Table 1: the sequences of PCR primers. Supplementary Table 2: the sequences of siRNAs. Supplementary Figure 1: the synthesis of recombinant RNLS and its role in the liver without exposing to IR. Supplementary Figure 2: the protective effect of NAD+ on oxidative stress in steatotic HepG2 cells. [file 7534285.f1.docx]

Supplementary table 1. The sequences of PCR primers.

Supplementary table 2. The sequences of siRNAs.

| SiRNA Targets | Sequences (5’-3’) |
| --- | --- |
| si-RNLS1# | GCUCGCCUAUUGAAGGAAUTT |
|  | AUUCCUUCAAUAGGCGAGCTT |
| si-RNLS2# | GGAACACAGCAUUGAGGAUTT |
|  | AUCCUCAAUGCUGUGUUCCTT |
| si-STAT31# | GCAACAGATTGCCTGCATT |
| si-STAT32# | CCACTTTGGTGTTTCATAA |
| si-NC | UUCUCCGAACGUGUCACGUUU |

| Primer set | Primers | Sequence (5’-3’) |
| --- | --- | --- |
| Homo-GAPDH | Forward | ACAACTTTGGTATCGTGGAAGG |
|  | Reverse | GCCATCACGCCACAGTTTC |
| Homo-RNLS | Forward | GCCTCTAAGCTCGCCTATTGAAGG |
|  | Reverse | GTAGCTCACAGCCTCCAGTTGC |
| Homo-STAT3 | Forward | TAACATTCTGGGCACGAACA |
|  | Reverse | GGCATCACAATTGGCACGG |
| Mmu-β-actin | Forward | CTGTCCCTGTATGCCTCTG |
|  | Reverse | ATGTCACGCACGATTTCC |
| Mmu-RNLS | Forward | AGTGAACGCCAGAGGGAGCAA |
|  | Reverse | TAGCGGCAGGACCAAGGGAC |
| Mmu-STAT3 | Forward | ACCTCTACCCCGACATTCCC |
|  | Reverse | GACATCGGCAGGTCAATGGTA |
| Mmu-ND1 | Forward | ACGCTTCCGTTACGATCAAC |
|  | Reverse | ACTCCCGCTGTAAAAATTGG |


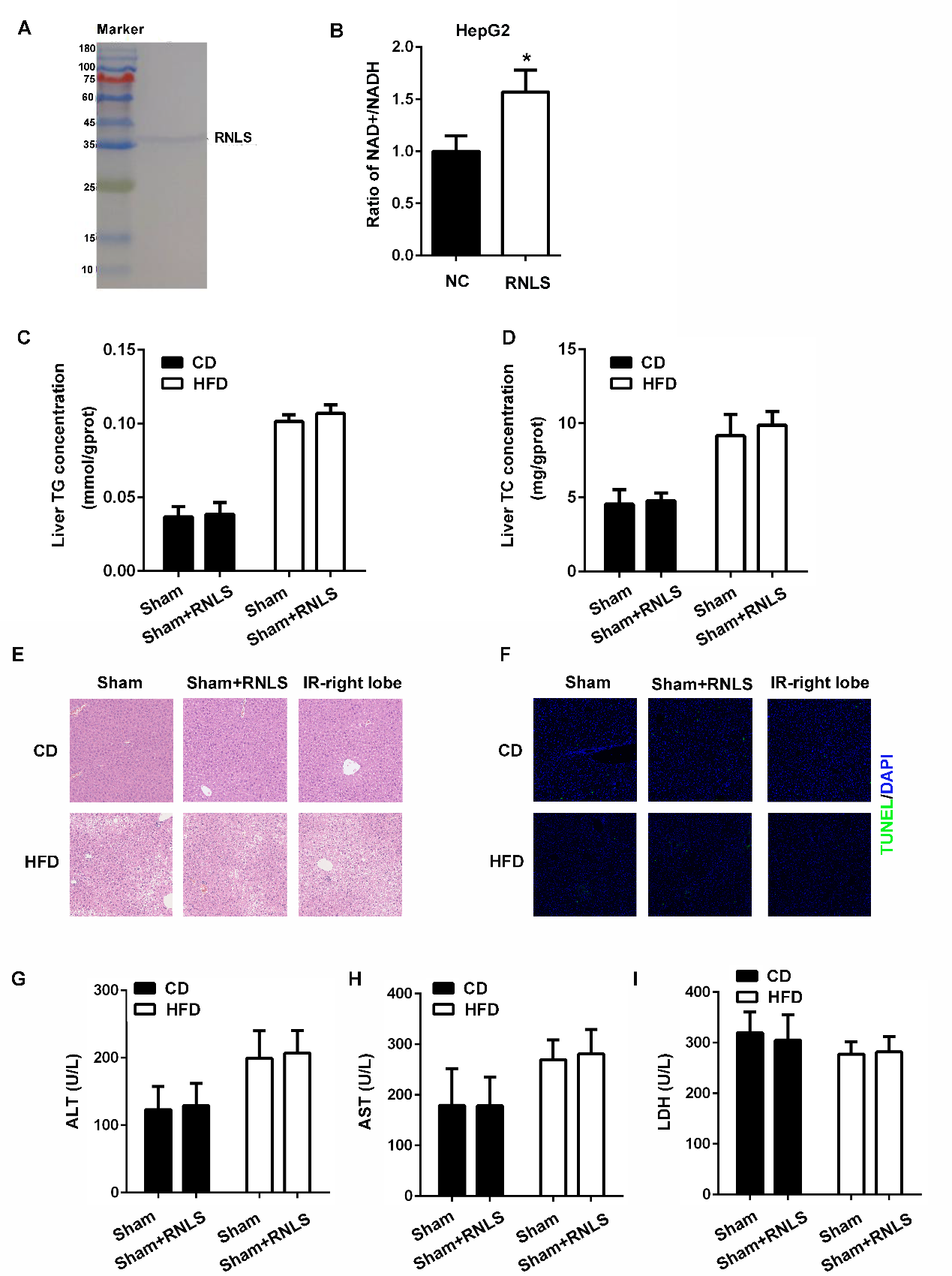


Supplementary figure 1: The synthesis of recombinant RNLS and its role in liver without exposing to IR. (A) Western blot analysis of recombinant RNLS after purification. (B) The enzymatic activity of recombinant RNLS detected by NAD^+^/NADH assay kit. TG (C) and TC (D) contents of mice liver form CD and HFD groups treated with or without RNLS. The liver of the Sham, Sham+RNLS, and IR-right lobe form CD and HFD groups were subjected to HE staining (E) and TUNEL staining (F). Serum ALT (G), AST (H), and LDH (I) levels form CD and HFD groups treated with or without RNLS. Data are plotted as the mean ± SD from three independent experiments.


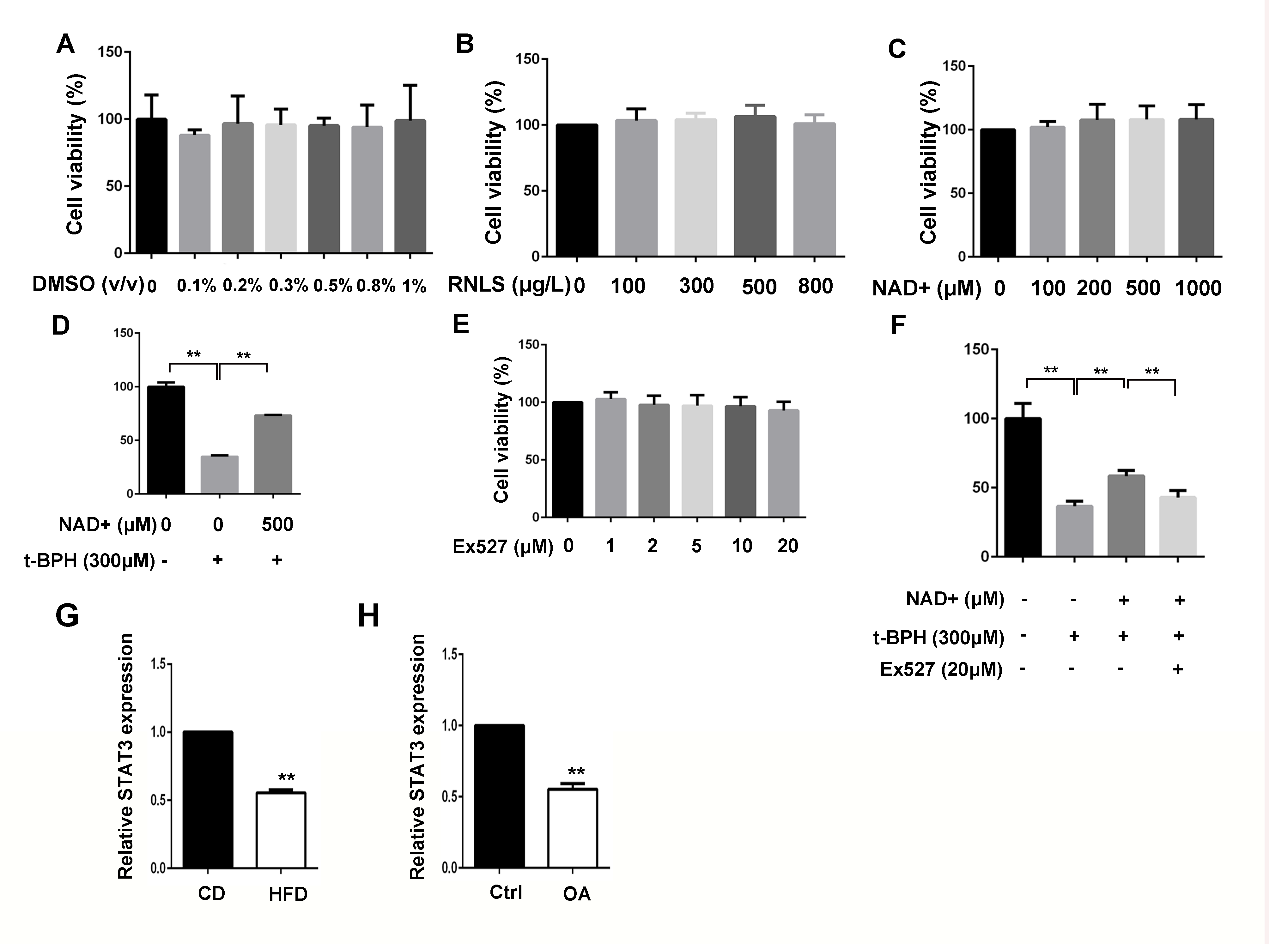


Supplemental figure 2: Supplemental figure 2: The protective effect of NAD^+^ on oxidative stress in steatostic HepG2 cells. Cytotoxicity test of DMSO (A), RNLS (B), NAD^+^ (C), and Ex527 (E). Cell viability of OA induced steatosis HepG2 cells pretreated with 500 μM NAD^+^ followed by 300 µM t-BHP treatment(D). Cell viability of OA induced steatosis HepG2 cells pretreated with NAD^+^ followed by 300 µM t-BHP treatment with or without EX527 administration (F). The mRNA level of STAT3 in livers (G) and HepG2 cells (H). **P<0.01. Data are plotted as the mean ± SD from three independent experiments.
